# Meta-Analysis of Relationship of Sleep Quality and Duration With Risk of Diabetic Retinopathy

**DOI:** 10.3389/fendo.2022.922886

**Published:** 2022-06-22

**Authors:** Zhenzhen Zheng, Chaoyu Wang, Chunhe Li, Qinglan Wu, Xiaojuan Chen, Huimin Chen, Huizhao Liao, Jinru Zhu, Junyan Lin, Xudong Ou, Zhihong Zou, Jinhua Liang, Riken Chen

**Affiliations:** ^1^Department of Respiratory and Critical Care Medicine, The Second Affiliated Hospital of Guangdong Medical University, Zhanjiang, China; ^2^Department of Respiratory and Critical Care Medicine, Taishan Hospital of Traditional Chinese Medicine, Jiangmen, China; ^3^Department of Critical Care Medicine, The First Affiliated Hospital of Guangzhou University of Chinese Medicine, Guangzhou, China; ^4^Department of Respiratory and Critical Care Medicine, Central People’s Hospital of Zhanjiang, Zhanjiang, China; ^5^Medical College, Jiaying Uiversity, Meizhou, China; ^6^Guangzhou Medical University, State Key Laboratory of Respiratory Disease, National Clinical Research Center for Respiratory Disease, Guangzhou Institute of Respiratory Health, The First Affiliated Hospital of Guangzhou Medical University, Guangzhou, China

**Keywords:** Pittsburgh sleep quality index, Epworth sleepiness scale, meta-analysis, diabetic retinopathy, sleep duration, sleep quality

## Abstract

**Objective:**

A meta-analysis is used to explore the relationship of sleep quality and duration with the risk of diabetic retinopathy (DR).

**Method:**

Cochrane Library, PubMed, Embase, and other databases are searched from their establishment to April 2022. Literature on the relationship of sleep quality and duration with DR risk published in various databases is collected, and two researchers independently screen the literature, extract data, and evaluate the quality of the included articles. The meta-analysis is performed with Review Manage 5.4.1 software.

**Results:**

A total of 7 articles are selected, including 4,626 subjects. The results show a strong correlation between sleep quality and DR risk. When comparing the sleep quality scores of “DR” (experimental group) and “NO DR” (control group), the Pittsburgh sleep quality index(PSQI) score of the DR group is significantly higher than that of the NO DR group (MD = 2.85; 95% confidence interval [CI] 1.92, 3.78, P<0.001), while the ESS score of the DR group is also significantly higher than that of the NO DR group (MD = 1.17; 95% confidence interval [CI] 0.14 to 2.30, P=0.04), so the sleep quality score of the DR group is higher than that of the NO DR group in both the PSQI and ESS scores, which confirms that low sleep quality is a risk factor for DR. Long sleep duration is also associated with the risk of developing DR; the number of adverse events (DR prevalence) is higher for “long sleep duration” than “normal sleep duration” [OR = 1.83, 95%CI 1.36–2.47, P < 0.001], suggesting that long sleep duration can cause increased DR risk. Short sleep duration is also associated with the occurrence of DR [OR = 1.49, 95%CI 1.15–1.94), P = 0.003] and can increase DR risk.

**Conclusion:**

Sleep quality and duration (including long and short sleep duration) are significantly associated with DR. To reduce DR risk, sleep intervention should be actively carried out, lifestyle changes should be made, and attention should be paid to the role of DR management.

Diabetes mellitus is one of the most common and frequently-occurring diseases in the world, and its rapidly rising prevalence has become a global public health problem. According to the International Diabetes Federation (1), there will be 700 million diabetes patients worldwide by 2045. Diabetic retinopathy (DR) is a serious complication of diabetes which may be caused by preventable insomnia in working-age people (2). Every year in the United States alone, 12,000 to 24,000 patients are blinded by DR (3), placing a heavy burden on individuals and society. Its incidence is related to the course of diabetes, age of onset, genetic factors, and control, and the longer the course of the disease, the higher the incidence. Most patients develop DR 10 to 15 years after the diagnosis of diabetes (4). Early intervention can effectively prevent the occurrence and development of DR, among which lifestyle intervention is the most cost-effective measure (5). Sleep disturbances have been found to be associated with abnormal glucose metabolism and an increased risk of diabetes (6). Sleep disturbances may be due to decreased sleep duration and/or decreased sleep quality (7). At present, the amount of clinical literature on the relationship of sleep quality and duration with DR is increasing year by year. However, there is a lack of randomized controlled trials with large multicenter samples, and few such evidence-based medical articles have been published in the world. Herein, a meta-analysis of observational studies on the relationship of sleep quality and duration with DR risk is carried out in order to provide medical evidence for the prevention and intervention of DR.

## Materials and Methods

### Retrieval Strategy

Cochrane Library, PubMed, Embase, and other databases were searched by computer from their establishment to April 2022. English search terms included “sleep quality”, “sleep wake disorders”, “sleep score”, “Pittsburgh sleep quality index”, “PSQI”, “Epworth Sleepiness Scale”, “ESS”, “sleep time”, “sleep duration”, “sleep amount”, and “diabetic retinopathy”.

### Literature Inclusion and Exclusion Criteria

Inclusion criteria: ① The subjects of the study were diabetic patients. ② The study was a cohort study, case-control study, or cross-sectional study. ③ The exposure factors were sleep quality score and sleep duration, wherein sleep quality score included the Pittsburgh sleep quality index(PSQI) and Epworth Sleepiness Scale (ESS) scores, and sleep duration included long sleep duration and short sleep duration. ④ The outcome indicators included mean difference (MD), odds ratio (OR), and 95% confidence interval (CI) after multivariate adjustment. ⑤ Based on different reports on the same research population, the studies with the largest sample sizes were included.

Exclusion criteria: ① Languages other than English. ② Studies for which the effect sizes could not be extracted or calculated. ③ Studies for which the original author did not respond or could not provide meta-analysis data.

### Literature Screening, Quality Assessment, and Data Extraction

Two researchers independently searched, selected, and screened the literature, then checked each other’s work and provided articles with differences to a third researcher for analysis to decide whether they should be included. The evaluation was performed using the “Risk of Bias Assessment” tool recommended by the Cochrane Collaboration, which is divided into three levels: low risk, unclear, and high risk. The contents are: whether it is randomly assigned; whether to carry out allocation concealment; whether to use blinding; whether the outcome data is complete; whether the research results are selectively reported; and whether there is any other risk of bias. The extracted data included the first author, study area, publication time, study type, sample size, age, PSQI, ESS, sleep duration assessment method, sleep duration grouping, outcome measures, and adjustment for confounders.

### Data Extraction and Definitions

The sleep quality scores of the research subjects were obtained by the PSQI and ESS questionnaires, and sleep duration was obtained by survey visits or questionnaires. DR was diagnosed by ophthalmologists through the analysis of fundus photos or conducting indirect ophthalmoscopy. In the included studies, DR included mild, moderate, severe, and any DR. The PSQI and ESS scoring standards of the sleep quality score were fixed and consistent, while the grouping and definition of sleep duration were not completely consistent. Long sleep duration was defined as > 8–9h, normal sleep duration as 6–8h, and short sleep duration as < 5–6h, all at night and within a 24h period.

### Statistical Methods

Statistical analysis was performed using Review Manage 5.4.1 software. MD and OR values were used for effect evaluation, and 95%CI was calculated. The heterogeneity of the studies was analyzed using the I^2^ statistic test and Q test. When I^2^ < 50% and P > 0.1, this indicated that there was no significant heterogeneity among the studies; when I^2^ > 50% and P < 0.1, this indicated that there was statistical heterogeneity among the studies. If there was still heterogeneity, a random effect model was used, and if there was no obvious heterogeneity, a fixed effect model was used. Sensitivity analysis was used to judge the stability and reliability of the combined results. P < 0.05 indicated that the difference was statistically significant.

## Results

### Literature Screening Results

**Figure 1:**
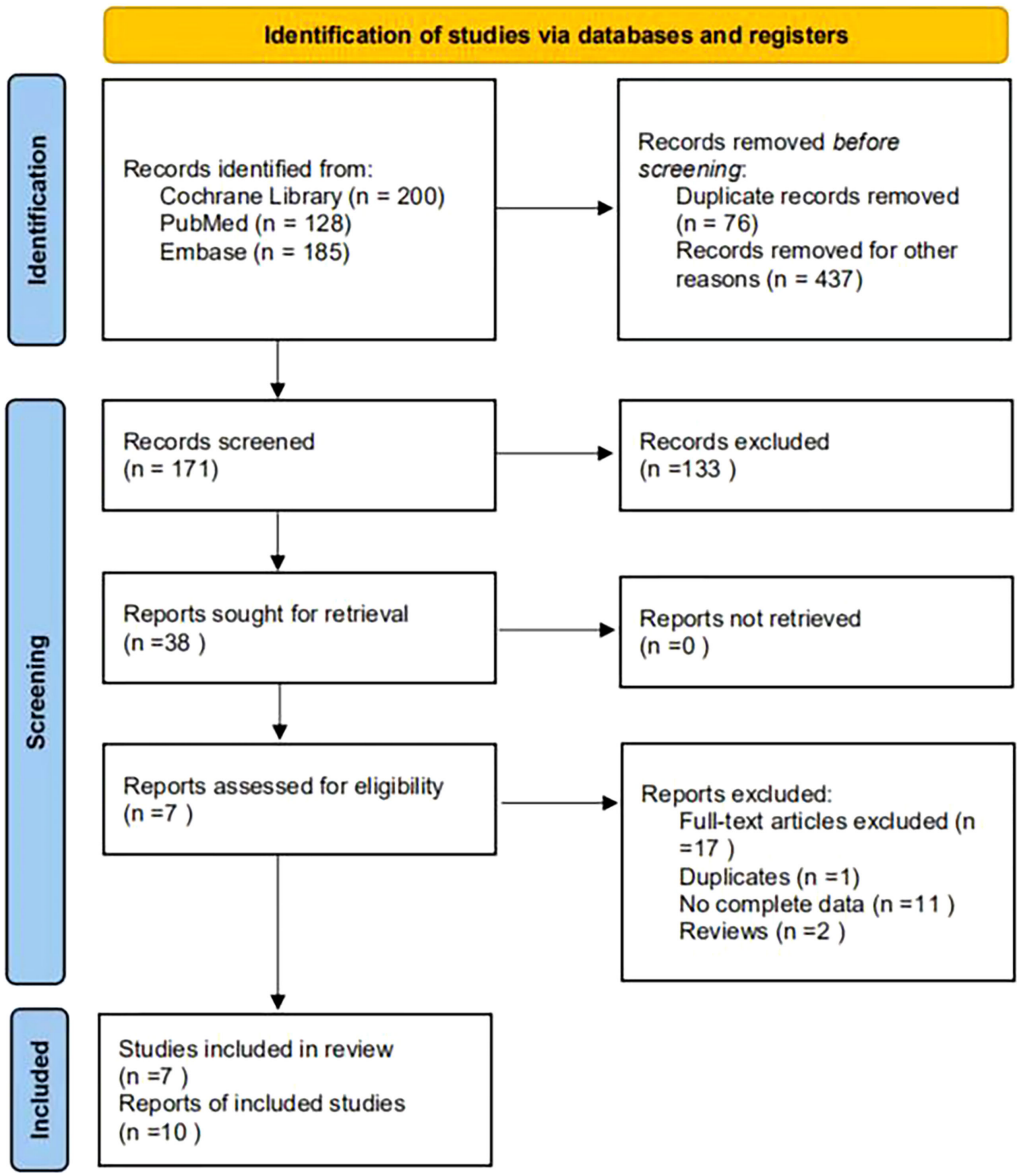
Flow diagram of literature screening.

A total of 513 articles were retrieved. A total of 437 papers were obtained after deduplication, and a total of 266 papers were excluded by reading the titles and abstracts. After reading the full texts, 7 papers were finally included (**Figure 1**), with a total of 4,626 research subjects. The study populations were from Korea, Singapore, India, Malaysia, Thailand, and Denmark. The basic characteristics of the articles included in the study are shown in **Table 1**.

**Table 1:** Characteristics of the included studies.

Study	Country	Sample Size	Mean age/year	Exposure assessment	Sleep duration/h	Global score (ESQI)	Global score (ESS)	Outcome	NOS score
Jee (8) 2017	Korean	1670	60.3	self-report	short(≦5) Long(≧9)	Not mentioned	Not mentioned	Any DR	7
Chew (9) 2020	Singapore	92	57.6+-8.3	self-report	short(<5)	Not mentioned	NO DR:4.8±2.5 DR:5.7±3.6	Moderate DR	7
Raman (10) 2012	India	1414	M:56.9±10.5 F:56.4±-8.9	self-report	short(<5) Long(>9)	Not mentioned	Not mentioned	Any DR	6
Tan (11) 2018	Malay India	1231	64.4±9.0	self-report	short(<6) Long(≧8)	Not mentioned	Not mentioned	Moderate DR	9
Dutta (7) 2020	India	140	NO DR:55.59±6.83 DR:57.51±5.82	self-report	Not mentioned	NO DR:4.30±3.26 DR:7.44±3.99	Not mentioned	Any DR	7
Sirisreet (12) 2020	Thailand	25	NO DR:51.1±11.5 DR:51.5±8.5	self-report	Not mentioned	NO DR:4.1±3.1 DR:7.4±3.5	Not mentioned	Any DR	7
Ba-Ali (13) 2018	Denmark	54	NO DR:63.1±8.3 DR:61.6±9.1	self-report	Not mentioned	NO DR:5.3±3.9 DR:7.2±4.5	NO DR:5.6±3.1 DR:7.2±3.6	Any DR	8

### Publication Bias and Sensitivity Analysis

**Figure 2:**
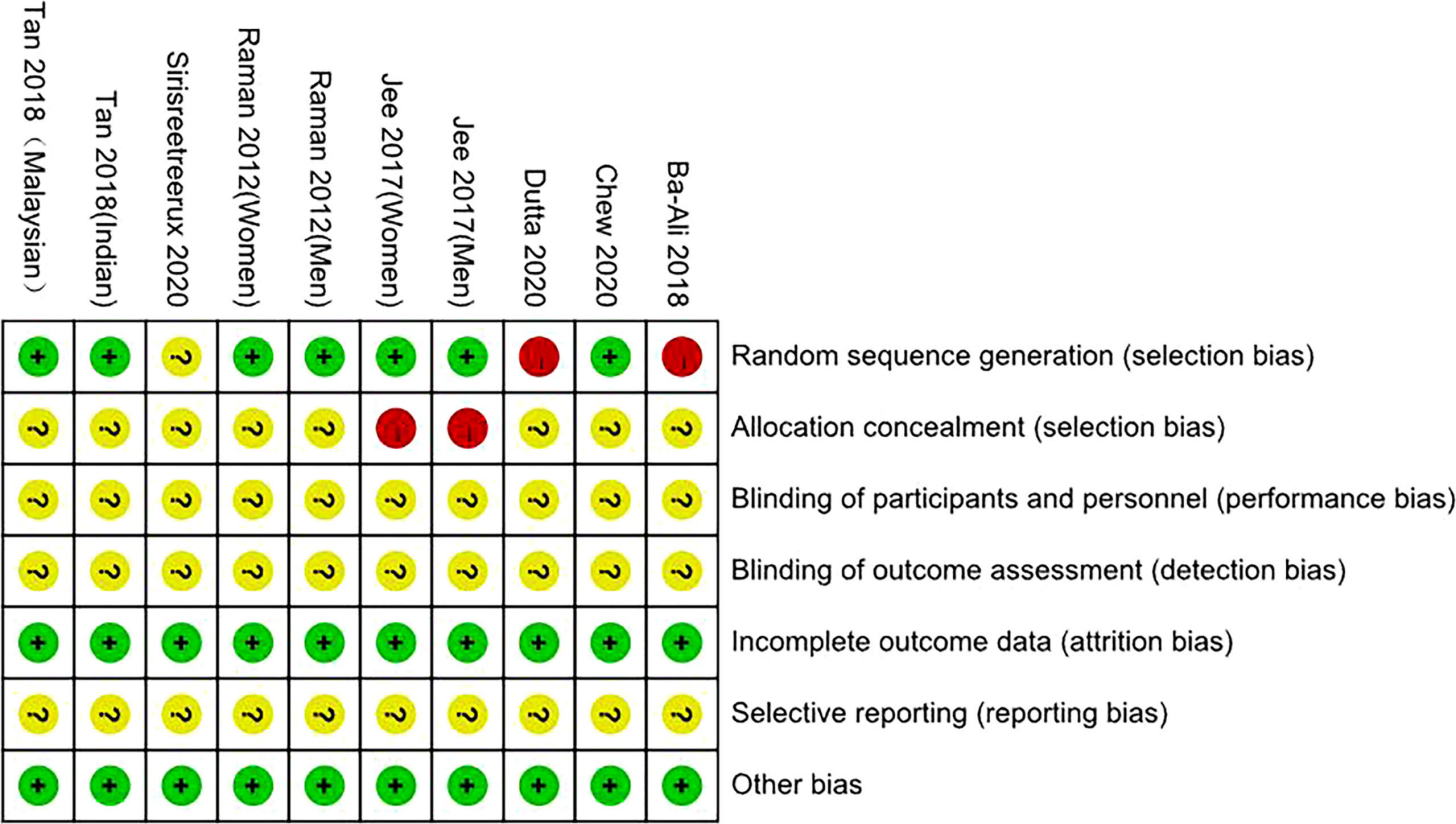
Overview of bias risk assessment of included literature.

**Figure 3:**
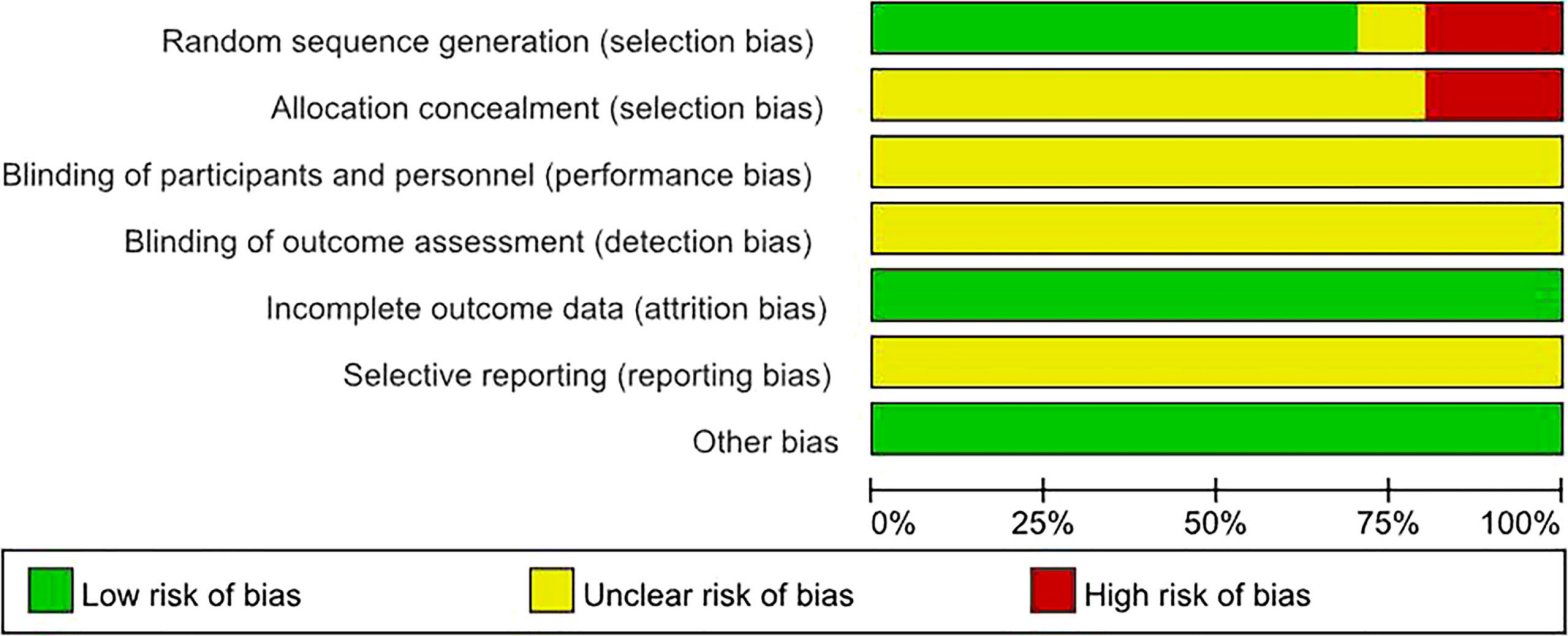
Bias risk assessment chart of included literature.

**Figure 4:**
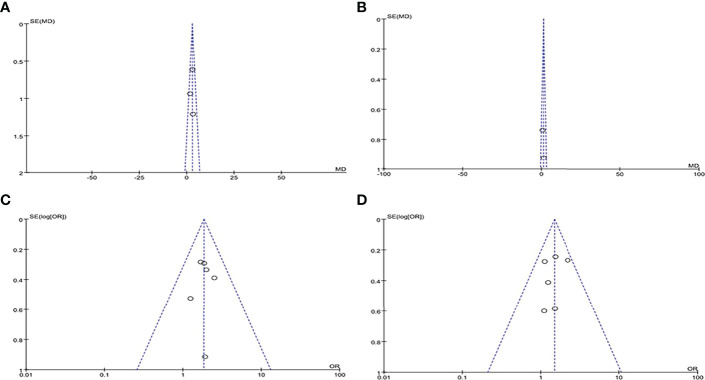
Funnel plots of included literature: **(A)** PSQI score funnel plot; **(B)** ESS score funnel plot; **(C)** Long sleep duration DR funnel plot; **(D)** Short sleep duration DR funnel plot.

By drawing funnel plots (**Figure 2**), the distribution of each study is shown to be basically symmetrical without any serious publication bias. The included studies showed a low risk of bias overall (**Figures 3**, **4**). Specifically: (1) two articles used the classification of subjects; (2) two articles did not have a clear degree of the non-concealment of subjects; (3) none of the articles mentioned blinding; (4) none of the articles mentioned the outcome indicators of selective reporting, and it was unclear whether there was any selective reporting of research results; (5) all articles and research data were complete.

The results of the sensitivity analysis showed that for DR risk in diabetic patients, all included studies had I^2^ < 50% and P > 0.1, meaning they were all homogeneous, so a fixed effect model was used. Among the three studies (PSQI score study, long sleep duration study and short sleep duration study), any articles that had no significant effect on the combined effect size of the remaining literature were removed to confirm the stability of the results; for the ESS score study, only two articles were included, and the sensitivity analysis was not carried out for two studies, so the results of the ESS score analysis were unstable.

### Statistical Analysis Results

#### Relationship Between Sleep Quality and DR Risk

**Figure 5:**
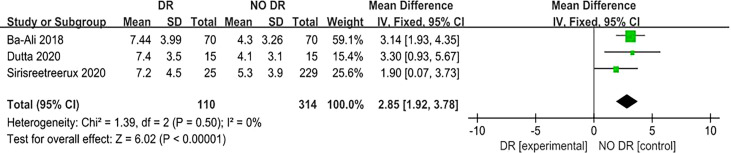
Forest plot of PSQI score and DR risk relationship meta-analysis.

**Figure 6:**
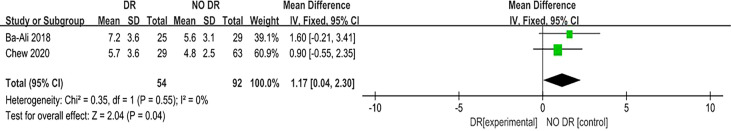
Forest plot of ESS score and DR risk relationship meta-analysis.

A total of three (7, 12, 13) articles reported that the PSQI score for sleep quality revealed its relationship with DR risk. There was no homogeneity among the studies (P = 0.50, I^2^ = 0%), so the fixed effect model was used. The results of the meta-analysis showed that the PSQI score of the diabetic population in the DR group (experimental group) was significantly higher than that in the NO DR group (control group), and the difference was statistically significant (MD = 2.85, 95%CI [1.92, 3.78], Z = 6.02, P < 0.00001), suggesting that the PSQI score is associated with DR risk and is a risk factor for DR. (**Figure 5**). Another two (9, 13) articles reported that the ESS score for sleep quality revealed its relationship with DR risk. There was no homogeneity among the studies (P = 0.55, I^2^ = 0%), so the fixed effect model was also used. The results of the meta-analysis showed that the ESS score of the diabetic population in the DR group (experimental group) was significantly higher than that in the NO DR group (control group), and the difference was statistically significant (MD = 1.17, 95%CI [0.04, 2.30], Z = 2.04, P = 0.04), suggesting that the ESS score is associated with DR risk and is also a risk factor for DR (**Figure 6**). Therefore, in both the PSQI and ESS scores, the sleep quality score in the DR group was higher than that in the NO DR group, confirming that sleep quality is related to DR risk and is a risk factor for DR.

#### Relationship Between Sleep Duration and DR Risk

##### Long Sleep Duration and DR Risk

**Figure 7:**
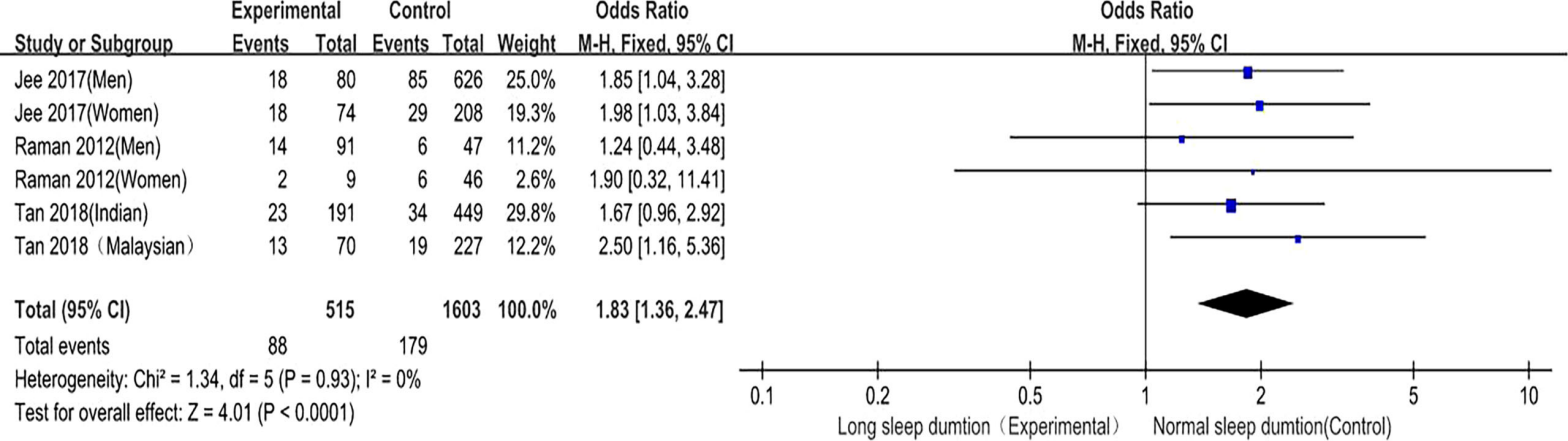
Forest plot of long sleep duration and DR risk relationship.

Long sleep duration and DR prevalence were included in 3 studies (8, 10, 11), and six studies provided the OR values and 95%CI of DR in diabetic patients in the long sleep duration group and control group. There was no heterogeneity among the studies (P = 0.93, I^2^ = 0%), so the fixed effect model was used. The meta-analysis results showed that the fixed effect model was (OR = 1.83, 95%CI 1.36–2.47, P < 0.0001), suggesting that compared with patients with normal sleep duration, the prevalence of DR in diabetic patients with long sleep duration was significantly higher (**Figure 7**). This confirms that long sleep duration is related to DR risk and is a risk factor for DR.

##### Short sleep duration and DR risk

**Figure 8:**
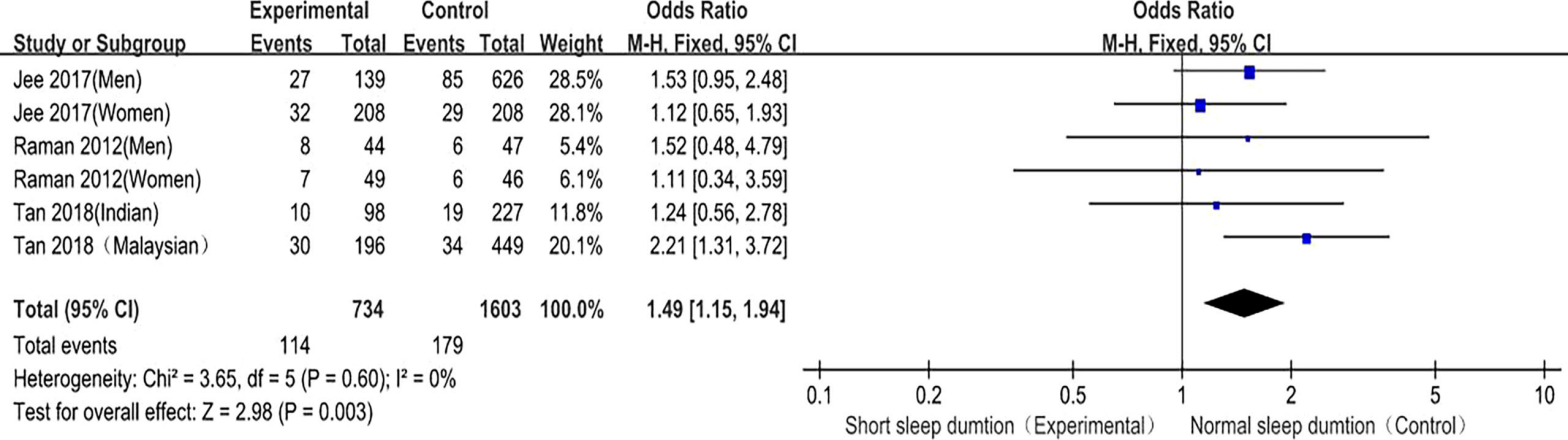
Forest plot of short sleep duration and DR risk relationship meta-analysis.

Short sleep duration and DR prevalence were included in 3 studies (8, 10, 11), and 6 studies provided the OR values and 95%CI of the incidence of DR in diabetic patients in the short sleep duration experimental group and normal sleep duration control group. There was no heterogeneity among the studies (P = 0.60, I^2^ = 0%), so the fixed effect model was used. The meta-analysis results showed that the fixed effect model was (OR = 1.49, 95%CI (1.15, 1.94), P = 0.003), suggesting that compared with patients with normal sleep duration, the prevalence of DR in diabetic patients with short sleep duration was significantly higher (**Figure 8**). This confirms that short sleep duration is related to DR risk and is a risk factor for DR.

## Discussion

Given the rising prevalence of diabetes and the high burden of DR worldwide, the prevention and treatment of DR is an increasing public health challenge (14). Therefore, in addition to classic risk factors such as poor glycemic control, hypertension, and longer disease duration, it is important to identify other modifiable risk factors from clinical and public health perspectives (15). The increasing prevalence of poor sleep health, which shows a long-term trend as modern society changes, is an underestimated determinant of health outcomes. A recent accumulation of evidence from experimental and epidemiological studies has shown that sleep duration and quality are significantly associated with diabetes, insulin resistance, and poor glycemic control (16–19). Our findings suggest that both sleep quality and duration may have an impact on the presence of DR.

“Sleep disturbances” refers to abnormal sleep quality, such as difficulty falling asleep, early awakening, and disturbances of the normal rhythm of sleeping and waking. Continuous hyperglycemia and blood sugar fluctuations in diabetes can cause damage to the cerebral blood vessels, nerves, cells, etc., and disrupt the nerves that regulate sleep-wake rhythms, and the series of diabetes symptoms or complications also result in sleep problems. Evidence suggests that sleep deprivation itself contributes to the pathogenesis of DR and the development of proliferative DR. Another consideration is that sleep disturbances have been shown to negatively impact immune function and inflammation (20, 21), the latter of which is implicated in the pathogenesis of DR (22) and is a target for drug therapy in DR (23, 24). At present, there are many ways to evaluate sleep quality, including objective monitoring methods such as polysomnography and various subjective questionnaires such as PSQI (25), ESS (26), insomnia severity index (ISI) (27), Leeds sleep evaluation questionnaire (LSEQ) (28), etc. In this study, sleep quality was assessed by PSQI and ESS scores. The higher the score, the worse the sleep quality. PSQI was proposed by Buysse et al. (25) in 1989. The reliability test of PSQI showed that the scale has good internal consistency (Cronbach’s alpha = 0.83) and test-retest reliability (r = 0.85). PSQI was developed on the basis of the analysis and evaluation of various scales related to assessing sleep quality. By organically combining the quality and quantity of sleep, it can not only evaluate the sleep behavior and habits of the general population, but also be used for the comprehensive evaluation of sleep quality in clinical patients (29). ESS item scores have been shown to be reliable, associated with sleepiness in everyday life, and effective in the assessment of sleep disturbances (30).

Through the analysis of the literature, it was found that more articles used the PSQI and ESS scores because they have good reliability and validity. Therefore, this study finally selected the PSQI and ESS scores as the outcome indicators. The PSQI and ESS scores of the DR experimental group were higher than those of the NO DR control group, meaning that sleep quality is significantly associated with DR and is a risk factor for DR. People with diabetes and poor sleep quality have a greater DR risk. Therefore, for DR patients with sleep disorders, in addition to specific problems that require symptomatic treatment, health education should be carried out at the same time, and clinical psychological and treatment intervention should be actively carried out to improve the sleep quality of patients, thereby improving their blood sugar control levels. Reducing the occurrence of diabetic complications and improving the quality of life of diabetic patients is of great significance.

A number of recent studies have found a relationship between sleep duration and diabetes risk (17) and its associated complications (10). The results of this study show that abnormal sleep duration, including long sleep duration and short sleep duration, is related to the occurrence of DR. Regarding the possible mechanism of the association between long sleep duration and DR: first, long sleep duration may be an indirect response of poor sleep quality, causing patients to require prolonged sleep; for example, obstructive sleep apnea is a known cause of increased sleep demand, and the resulting intermittent hypoxia may play an important role in the development of diabetes, and has been identified as a risk factor for insulin resistance and diabetes (16). Second, as with short sleep duration, long sleep duration also increases inflammatory biomarkers (31), and pro-inflammatory cytokines have sleep-inducing effects, so prolonged sleep may also be induced by diabetes itself and/or the resulting inflammatory states (32). Third, in a dark environment, the oxygen consumption of the outer retina increases, which leads to a sharp decrease in the retinal oxygen tension curve, aggravates retinal hypoxia, and stimulates the overproduction of vascular endothelial growth factor, which in turn promotes the occurrence of DR (33, 34). Fourth, long sleep duration is often clustered with other known risk factors for DR such as depression, low socioeconomic status, history of other medical diseases, and less physical activity (35, 36).

Short sleep duration may also affect the progression of DR through various mechanisms. First, short sleep duration is associated with poor glycemic control (37). Buxton et al. showed that sleep deprivation leads to decreased insulin sensitivity (18) and affects insulin secretion through the neuroendocrine system (38). Second, short sleep duration may be associated with inflammation (21). Third, short sleep duration may lead to vascular endothelial dysfunction by affecting nitric oxide, serum endothelin, etc. (39, 40). Fourth, sleep disruption affects melatonin levels (41), accelerating the progression of DR. At the same time, similar to long sleep duration, short sleep duration is often clustered with other DR-related risk factors such as low socioeconomic status and low education level (36).

It should be noted that DR itself also affects sleep duration and quality (42). Especially in elderly patients, the presence of diabetic complications is associated with depression and sleep duration (43). As there were insufficient studies on the association between DR and sleep stage, the relationship between the two could not be further investigated. Therefore, the causal relationship between the two remains to be determined through further research.

This study has several limitations: first, the sleep quality score was obtained in the form of a questionnaire, moreover, the results of the ESS score studies are not stable, the number of included studies is small, more studies are needed to further stabilize the study results; and sleep duration was mainly reported by patients, which is highly subjective. Moreover, the definitions of long and short sleep duration were not completely consistent among studies. Second, the methods of diagnosing and grading DR in each study were slightly different, and the study populations were from different ethnicities, which may have led to greater heterogeneity in the results. Third, the sample size was relatively small, and there was a lack of large-scale, multi-center, randomized controlled trials. Fourth, the quality of some included articles was not very high, so there may be selection bias. Therefore, the conclusions of this study should be interpreted with caution.

In conclusion, Our results suggest that both sleep quality and duration are related to the occurrence of DR. Among them, sleep quality and duration are both risk factors for DR, while the relationship between short sleep duration and DR needs to be further determined, and its causal relationship and biological mechanism also require further research for confirmation. Sleep quality and duration, as individual-level modifiable risk factors, should be of great concern to clinicians and patients.

## Data Availability Statement

The original contributions presented in the study are included in the article/supplementary material. Further inquiries can be directed to the corresponding author.

## Author Contributions

ZZZ, CW, CL, QW and XC are the guarantor of the manuscript and take responsibility for the content of this manuscript. JHL, ZZZ, RC and CW contributed to the design of the study. HC, JZ, JYL and XO were involved in the data analysis. ZHZ, XO and RC contributed to the acquisition of primary data. ZZZ, CW and RC wrote the initial draft of the manuscript. ZZZ, RC and JHL contributed significantly to the revision of the manuscript. All authors read and approved the final manuscript.

## Funding

This study was funded by the Natural Science Foundation of Guangdong Province (2019A1515010981).

## Conflict of Interest

The authors declare that the research was conducted in the absence of any commercial or financial relationships that could be construed as a potential conflict of interest.

## Publisher’s Note

All claims expressed in this article are solely those of the authors and do not necessarily represent those of their affiliated organizations, or those of the publisher, the editors and the reviewers. Any product that may be evaluated in this article, or claim that may be made by its manufacturer, is not guaranteed or endorsed by the publisher.
